# A metabolic model of the leaf-associated bacterium *Paraburkholderia dioscoreae* links the ethylene precursor ACC to core carbon pathways

**DOI:** 10.1128/msystems.00514-26

**Published:** 2026-07-13

**Authors:** Pablo Rolle, Hannah Auricht, Andreea Marcut, Cristina López-Hidalgo, Wolfram Weckwerth, Steffen Waldherr

**Affiliations:** 1 Department of Functional and Evolutionary Ecology, Faculty of Life Sciences, University of Vienna 27258, Vienna, Austria; 2 Environment and Climate Hub (ECH), University of Vienna, Vienna, Austria; 3 Vienna Metabolomics Center (VIME), University of Vienna, Vienna, Austria; 4 Research Platform Artificial Intelligence for personalized nutrition, University of Vienna, Vienna, Austria; 5 Health in Society Research Network, University of Vienna, Vienna, Austria; Universiteit Leiden, Leiden, the Netherlands

**Keywords:** metabolic modeling, plant-bacteria interactions, flux balance analysis

## Abstract

*Paraburkholderia dioscoreae* MSB3^T^ is a novel species with potential agricultural impact, isolated from leaf acumens of the “air potato yam” *Dioscorea bulbifera. P. dioscoreae*’s genome encodes a relevant combination of features mediating a beneficial plant-associated lifestyle, and it exhibits significant growth promotion when applied to agriculturally important plants such as tomato. Here, we constructed iPR1691, the first genome-scale metabolic model for *P. dioscoreae* MSB3, which includes 1,687 reactions, 1,487 metabolites, and 1,691 genes. From an available annotation of *P. dioscoreae*’s genome, a draft model was constructed using an automated approach followed by automatic gap-filling on Kbase and was manually curated using constraint-based modeling. The plant growth-promoting effect is related to 1-aminocyclopropane-1-carboxylic acid (ACC), an ethylene precursor in plants, which *P. dioscoreae* can use as a carbon and nitrogen source. Based on the reactions and genes present in the model, and on various pathways available in the MetaCyc database, a pathway for ACC usage was reconstructed. A sequence comparison against a library of phyllosphere bacterial genomes showed a broad occurrence of the pathway genes in phyllosphere strains. Flux balance analysis was used to validate growth of *P. dioscoreae* on different substrates, corresponding to diverse conditions of carbon and nitrogen availability. The model provides a representation of bacterial growth on ACC, thus elucidating the biochemical and genetic mechanisms of this plant-bacterial interaction.

**IMPORTANCE:**

Metabolization of the plant ethylene precursor 1-aminocyclopropane-1-carboxylic acid (ACC) is an important mechanism for plant-associated bacteria to regulate their host’s ethylene status. While previous studies have typically focused on the first step (ACC deaminase) as a metabolic sink, we further investigated the metabolic role of ACC for the bacterium. Our model system is the species *Paraburkholderia dioscoreae*, isolated from leaf acumens of the “air potato yam” *Dioscorea bulbifera* and shown to mediate a beneficial plant-associated lifestyle, including significant growth promotion when applied to agriculturally important plants such as tomato. Here, we constructed the first genome-scale metabolic network reconstruction for *P. dioscoreae*, validated against *in vitro* growth data. The model incorporates a novel pathway that links ACC to succinate and thus explains the utilization of ACC as a carbon source. Moreover, a sequence similarity search of the pathway genes in a library of phyllosphere bacteria indicates that the pathway is present in several other leaf-associated species.

## INTRODUCTION

The effects of microbes on plants are an important aspect of plant physiology and growth, be it as plant pathogens that endanger the plant or as symbionts that may contribute to increased plant growth. Although different beneficial interactions of plants with diverse microbial communities have been known for a long time, much of the previous research was focused on root-associated microbes (the rhizosphere), while knowledge of the leaf-associated microbiome (the phyllosphere) has lagged behind. Only recently, the microbiology of leaf surfaces has gone beyond pathogen interactions to a broader understanding of the phyllosphere community composition and functional roles (1).

Here, we consider the phyllosphere bacterium *Paraburkholderia dioscoreae* MSB3, which is a newly discovered species with a plant growth-promoting effect, isolated from the leaf acumen of the “air potato yam” *Dioscorea bulbifera* (2). *P. dioscoreae*’s genome encodes a relevant combination of features mediating a beneficial plant-associated lifestyle that includes biological nitrogen fixation, plant hormone regulation, detoxification of xenobiotics, degradation of aromatic compounds, and multiple protein secretion systems (3). *P. dioscoreae* even exhibits significant growth promotion when applied to agriculturally important plants such as tomato, increasing total dry biomass by 40% (2), demonstrating the relevance of studying this organism and its properties.

One of the key mechanisms in growth-promoting bacteria, including *P. dioscoreae* MSB3, is the reduction of plant ethylene levels by the activity of the enzyme 1-aminocyclopropane-1-carboxylate (ACC) deaminase (4, 5). Ethylene is a potent growth regulator, associated with fruit ripening, growth arrest, and senescence (6–8), although a previous study (6) reported that at low concentrations, ethylene could be involved in growth stimulation. ACC is a direct metabolic precursor of ethylene but can be cleaved into α-ketobutyrate and ammonia by the ACC deaminase enzyme (9, 10), which is a possible way for phyllospheric bacteria to interfere with plant growth arrest and senescence. Since the ACC cleavage products are core carbon and nitrogen metabolites, they could potentially also serve as basic nutrients for the bacteria. In fact, Herpell et al. have shown that *P. dioscoreae* can grow on ACC without other carbon or nitrogen substrates (5).

To further explore this metabolic capability, we constructed the first genome-scale metabolic model (GSMM) for *P. dioscoreae* MSB3, iPR1691. GSMM reconstruction has become one of the major modeling approaches to systems biology, which computationally describes a network containing the metabolic reactions in an organism and the genes encoding for each enzyme based on genome annotation data (11, 12). As part of the model, a pathway for the consumption of ACC as a carbon and nitrogen source is proposed, built from present reactions in the model and information found in the MetaCyc database (13). With the integration of that pathway in a GSMM for *P. dioscoreae* MSB3, we aim to trace the involved enzymes and characterize the overall metabolic state where the bacterium is using ACC as a carbon and nitrogen source.

The model is validated against experimental data from growth on *P. dioscoreae* on different carbon substrates and ACC and reproduces the relative biomass yields on these substrates to a good accuracy. Furthermore, the proposed ACC pathway appears to be present in a considerable fraction of other phyllosphere bacteria, based on a computational comparison of a phyllosphere genome collection against the enzymes in the ACC pathway. Overall, the proposed model thus improves understanding of ACC breakdown and utilization by phyllosphere bacteria and can serve as a basis for further analysis of their interactions with host plant metabolism.

## MATERIALS AND METHODS

### Metabolic model reconstruction

The draft model construction and gap-filling were performed on a Kbase enviroment using the tools “MS2 - Build Prokaryotic Metabolic Models” and “MS2 - Improved Gapfill Metabolic Models,” which use a ModelSEED pipeline to construct a model based on input annotated genomes (14).

For the first step, the *P. dioscoreae* MSB3 genome annotation obtained from NCBI (NCBI RefSeq ID GCF_902459535.1) was given as input, and the option to apply ATP correction was activated. The “gram-negative” template was used for the reconstruction, and the corresponding template biomass reaction was retained as such in the model. Prior experimental data showed that *P. dioscoreae* can grow on glucose efficiently, and to ensure just the filling of a minimum set of reactions necessary for growth in this condition, gap-filling was performed with the initial draft model using the “carbol-D-glucose” medium (a glucose minimal medium) available on Kbase. The medium composition is presented in Table S1.

During the manual curation, FBA was performed using the cobrapy library (version 0.25.0) (15) with a maximization of the biomass reaction and ATP production as objectives, both with glucose as the carbon source. To reduce the impact of thermodynamically infeasible cycles, the reactions that had a flux value close to the default flux bound ±1,000 $\frac{mmol}{gDWh}$ were checked individually. For these reactions, the directionality was compared to the thermodynamic information and annotation in the MetaCyc database and corrected whenever the directionality in the model was not the same as in the database. An example is the reaction rxn00154_c0 (pyruvate:NAD + 2-oxidoreductase [CoA-acetylating], with BioCyc ID: PYRUVDEH-RXN, and EC number: 1.2.1.104), which produces acetyl-CoA from pyruvate, using NAD+/H as cofactor. In the draft model, it was reversible, with a flux of −997.89 $\frac{mmol}{gDWh}$, while in MetaCyc, it was annotated only in the forward direction. The procedure was iterated until no more excessive flux values were found.

Furthermore, to ensure that thermodynamically infeasible cycles did not affect the model results, we compared the flux distribution given by the standard FBA function in cobrapy [model.optimize()], in a glucose medium, with three different functions that ensure thermodynamic feasibility: the CycleFreeFlux algorithm (16), which takes the solution of the standard FBA and minimizes the sum of absolute fluxes, while keeping the same direction in all reactions, and the optimal value of the original objective function; the loopless formulation (17), which adds mixed-integer constraints to the model that serve to prevent any loops; and the parsimonious FBA (pFBA) approach (18), which uses a hierarchical optimization in which growth rate is maximized by standard FBA followed by a minimization of the sum of fluxes. In cobrapy, these methods are implemented as cobra.flux_analysis.loopless_solution, cobra.flux_analysis.add_loopless, and cobra.flux_analysis.pfba, respectively.

In addition to comparing the standard FBA solution to the various “cycle-free” solutions, a flux variability analysis (FVA) test was performed to identify reactions that could take part in cycles but did not have such high fluxes in the FBA solution. These reactions would have a minimum or maximum FVA flux close to or equal to ±1,000 $\frac{mmol}{gDWh}$.

The analysis for thermodynamically infeasible cycles was done for four different objective functions: maximization of biomass, ATP hydrolysis, as well as NADH and NADPH oxidation reactions, with the latter two only being added temporarily to the model.

Model validation results using the MEMOTE tool (19) are reported in the results based on the final curated version of the model, with the same glucose medium as used in gap-filling. MEMOTE also identifies mass and energy imbalances, and the reactions and metabolites that were found were corrected when possible, comparing with MetaCyc and KEGG databases (20).

The curated model is provided as an SBML file in Data set S1 and has been submitted to the BioModels database (21).

### Blast analysis of ACC pathway genes against *A. thaliana* leaf bacterial strains

The 59 genes associated with reactions in the proposed ACC consumption pathway were extracted from the model, and their amino acid sequence was obtained from the translation of the genomic sequences in the genome annotation. Each gene was blasted against a library of 224 bacterial genomes obtained from *A. thaliana* leaf microbiome samples, reported in a previous study (22), with a hit threshold of 25% sequence identity and an *e*-value of 10^−10^. In the literature, a percent identity of 30% is used as a rule of thumb and is considered conservative for statistically significant protein homology, with 25% giving more relevant homology hits, and an *e*-value under 10^−6^ is considered for statistical significance (23). In case of multiple hits, the highest scoring hit for each gene and strain is used to assemble Fig. 3.

### Flux balance analysis for different carbon substrates

Flux balance analysis was performed as pFBA in all the different analyses to ensure thermodynamic feasibility, using the cobrapy library. Unless otherwise mentioned, the optimization objective was to maximize the flux of the biomass-producing reaction, which corresponds to the growth rate $\mu [\frac{1}{h}]$ of the bacteria. Medium composition was accounted for by adjusting the constraints of the model’s exchange reactions for the respective medium compounds, for example, setting the limit for the intake of glucose to 5 $\frac{mmol}{gDWh}$ constrains the model to a consumption of glucose up to that value. While the chosen constraints yield growth rates of similar values as observed empirically (0.1 – 0.3 $\frac{1}{h}$; cf. Fig. S3), in the absence of more detailed uptake kinetics, we cannot quantify the uptake fluxes accurately. Thus, for the model analysis, we consider mostly yields, that is, ratios of production vs. uptake fluxes, which are commonly quite robust against variations in quantitative uptake fluxes in FBA constraints. The constraints based on the medium composition are reported in changing the carbon source and bounds accordingly, and in the case of ACC, setting the intake flow of ammonium (NH_3_) to zero.

### Biomass yields calculations for different substrates

Biomass yields were calculated by performing FBA under different carbon and nitrogen sources, and dividing the growth rate by the total carbon uptake. Glucose, succinate, malate, and ACC were used as carbon sources, with ammonium and ACC as nitrogen sources. In all cases, FBA was run with uptake constraints from 2 to 10 $\frac{mmol}{gDWh}$ on the corresponding exchange reactions, and the yields presented are calculated as an average of the yields for each uptake constraint. In the case of ACC being the nitrogen source, its uptake was constrained to a flux value in the ideal ratio to the considered other carbon source, that is, corresponding to the threshold where both ACC and the carbon source are limiting and no excess ammonium production occurs.

### Phenotypic phase plane (PhPP)

A phenotypic phase plane is the response surface formed by changing two parameters, for example, the uptake constraints of two different substrates, and computing the corresponding model prediction (growth rate). This yields a 3D surface where the growth rates are plotted above the (*x*, *y*) plane formed by the two considered parameters (24).

Phenotypic phase planes were plotted with a custom Jupyter Python notebook with the matplotlib library (25), for varying combinations of glucose vs. ammonia uptake constraints, and glucose vs. ACC uptake constraints, in both cases ranging from 0 to 50 $\frac{mmol}{gDW\;h}$. For glucose with ACC, we consider a first variant without ammonium in the medium (zero exchange flux), and a second variant with unconstrained ammonium availability (no bound on the exchange flux).

### Growth of *P. dioscoreae* on glucose and ACC medium for growth curves

*P. dioscoreae* MSB3 stocks stored at −80°C were thawed and inoculated into lysogeny broth (LB) medium under sterile conditions, followed by an overnight incubation at 28°C and shaking at 250 rpm to restore metabolic activity and support cellular recovery. All growth data were obtained using adapted M9 minimal medium (0.24 M disodium phosphate, 0.11 M potassium dihydrogen phosphate, 0.04 M sodium chloride, 0.002 M magnesium chloride, and 0.001 M calcium chloride) without ammonium chloride and glucose. This enabled us to trace growth of *P. dioscoreae* on different carbon and nitrogen sources. After harvesting the bacterial pellet from the LB medium culture, cells were washed three times with 20 mL of adapted M9 minimal medium to remove any remaining nutrient sources. Then, equimolar amounts (2 mM) of glucose and ammonium chloride or ACC were added to the adapted M9 medium. The prepared media were inoculated with washed *P. dioscoreae* at the above cultivation conditions in three replicates for each condition, and the optical density (OD_600_) of the culture was measured at 11 time points over a period of 150 h, except in the cases of growth in succinate and malate where, due to a fast initial growth, measurements were at eight time points in a period of 96 h.

### Yield comparison against experimental data

To compare the experimental results with the model, we normalized against the results obtained in a glucose + ammonium medium. For the experimental results, we randomly selected pairs of values (*a*, *b*) in stationary phase, where “*a*” is a value from the case analyzed and “*b*” is a value from the glucose + ammonium case. We ran 10^4^ iterations for every case, obtaining an average value of $\frac{a}{b}$ and standard deviation. For the model, we ran the model setting the fluxes for glucose, ACC, and ammonium with the same values as concentrations used in the experiments and divided the growth rate obtained by FBA against the growth in the glucose + ammonium media.

## RESULTS

### Construction of the metabolic model

An initial draft metabolic network model was constructed in the KBase environment using the ModelSEED2 pipeline (14, 26) from the publicly available genome sequence for *P. dioscoreae* (2). The biomass reaction is taken from ModelSEED’s template for gram-negative bacteria. The reconstruction pipeline includes gap-filling to ensure the availability of biosynthesis pathways for all biomass components and adds exchange reactions for possible substrates and metabolic by-products. The gap-filled reactions were mainly related to “ubiquinone and other terpenoid-quinone biosynthesis,” “porphyrin metabolism,” and some amino acids, cofactors, and vitamins biosynthesis, and/or degradation. Core pathways that were known to be present from the previous genome analysis (2) and relevant for *P. dioscoreae* physiology were verified manually and completed in the model where necessary. The reconstructed model was further analyzed with flux balance analysis in different media with the Python package cobrapy (15) for manual curation. In the manual curation, we verified and, where necessary, corrected the directionality of multiple reactions according to thermodynamic information in the MetaCyc database. In addition to directionality, some reaction equations and metabolite chemical formulas were corrected to ensure consistent mass balances. The full list of gap-filled reactions and of reactions and metabolites that were corrected or added through the manual curation is given in Data set S2.

**Fig 1:**
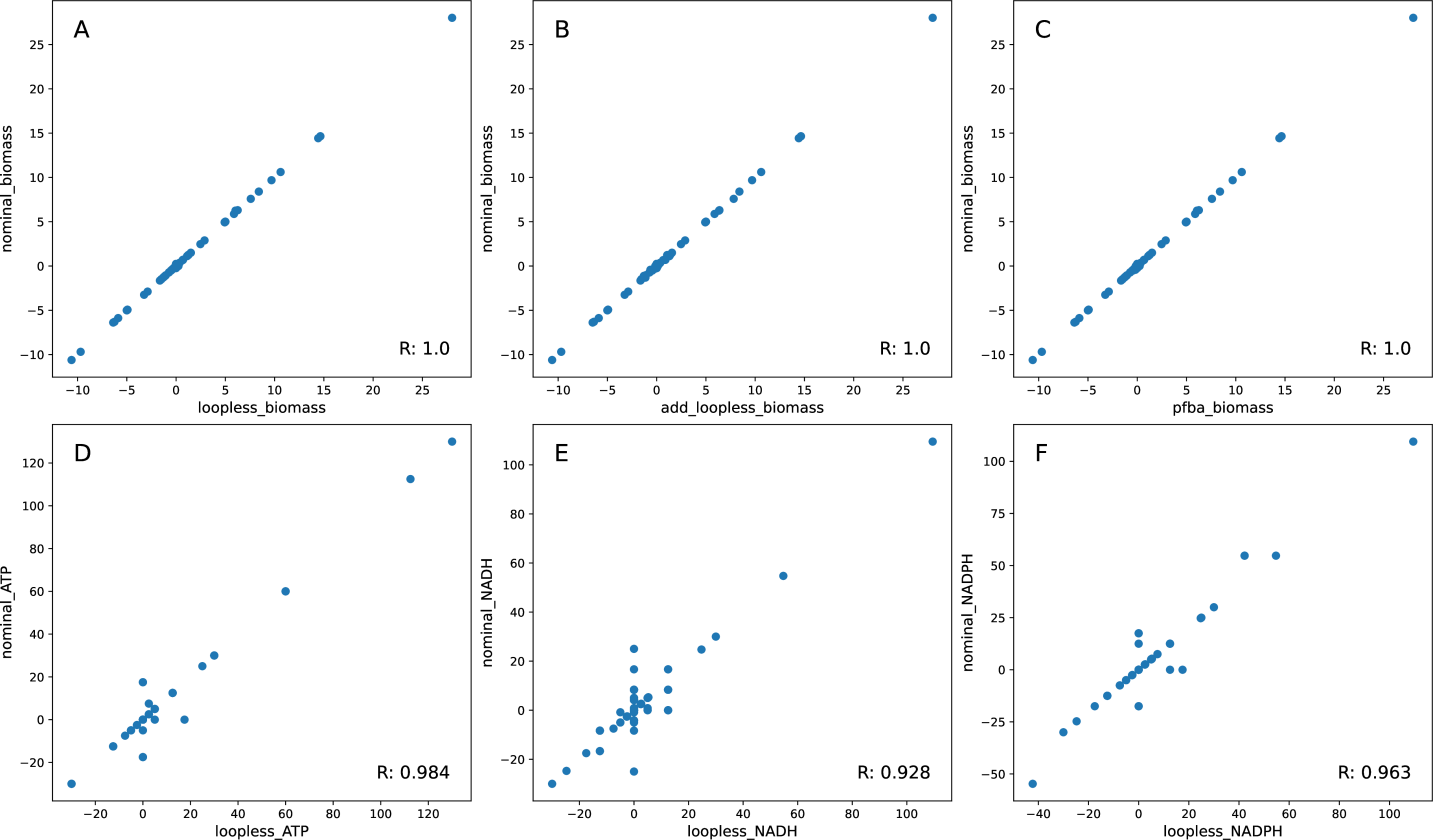
Comparison of standard FBA results against loopless approaches. (**A through C**) Comparison of fluxes maximizing biomass, where “nominal_biomass” are standard FBA fluxes, and “loopless_biomass,” “add_loopless_biomass,” and “pFBA_biomass” are loopless fluxes according to the different algorithms explained in Materials and Methods. (**D through F**) Comparison of fluxes maximizing energy metabolite production, where “nominal_ATP,” “nominal_NADH,” and “nominal_NADPH” are standard FBA fluxes, and “loopless_ATP,” “loopless_NADH,” and “loopless_NADPH” are loopless fluxes from the CycleFree algorithm.

To ensure that there were no thermodynamically infeasible cycles, we compared standard FBA, maximizing growth rate, with three analysis algorithms that ensure thermodynamic feasibility as outlined in the Materials and Methods section (Fig. 1A through 1C). With a Pearson correlation coefficient *R* consistently at 1.0, and no fluxes at the default bound of ±1,000 $\frac{mmol}{gDWh}$, the three approaches show that, when maximizing biomass production in glucose media even with standard FBA, there are no infeasible cycles in the optimal solution. The nominal distribution has little differences to the loopless ones, with some fluxes that are mirroring each other, suggesting alternative reactions.

We also compared the standard FBA solution with the CycleFree algorithm, maximizing ATP, NADH, or NADPH production (Fig. 1D through F). Similarly to the previous test, we obtained almost no difference between the two solutions, with an average Pearson correlation coefficient *R* of 0.958 between the three cases, and no outliers with extreme fluxes.

Although the nominal FBA results do not show infeasible cycles in the solution, we checked with FVA if there were reactions that could be taking abnormally high flux values in the solution space. We ran FVA in the same cases as before, maximizing biomass, ATP, NADH, or NADPH production. In all of the scenarios, we found the same 128 reactions that, in the solution space, could have an abnormally high flux, thus indicating that there can still be cycles present in the overall network. Since these cycles do not seem to appear when just optimizing for biomass, ATP, or NAD(P)H turnover, they would not contribute erroneously to biomass production or energy metabolism. Still, it is recommended to analyze the model with a method that minimizes spurious fluxes, such as parsimonious FBA, to avoid any impact of these cycles on the results.

To check completeness of the model annotation and formal consistency of the network, we applied the metabolic network validation software MEMOTE (19). In general, MEMOTE checks for model annotation in terms of gene, reaction, and metabolite identifiers being present, metabolites being annotated with chemical formulas and charge information, stoichiometric consistency of the model, and possible issues connected with metabolic cycles. Table 1 shows a summary of the network properties and MEMOTE verification results. A fraction of 78.1% of the reactions are internal reactions that are associated with at least one gene present in the genome annotation, and only 1.6% are reactions incorporated through gap-filling, which do not have a known associated gene in *P. dioscoreae*. The model is found to be stoichiometrically consistent without significant mass or charge imbalances. The overall achieved MEMOTE score is 89%, which is within the typical range of values for well curated models; for example, the recent iML1515 model, a highly curated model for *E. coli* K-12, has a MEMOTE score of 91% (27). The full MEMOTE report of the model in glucose media can be found in Data set S3.

**TABLE 1:** Key features of the reconstructed metabolic network model

Model feature	Value
Number of reactions	1,687
Internal reactions	1,348
Gap-filled reactions	27
Transport reactions	182
Exchange reactions	153
Number of metabolites	1,487
Number of genes	1,691
Reactions with GPR	1,471
Mass balance	99.70%
Charge balance	96.90%
Stoichiometric consistency	100%

### A newly reconstructed pathway links ACC consumption to succinate

The hallmark reaction for ACC degradation is ACC deaminase, which hydrolyzes ACC to ammonia and 2-oxobutyrate (9). ACC deaminase is present as the acdS gene in the genome of *P. dioscoreae* MSB3 and was shown to allow for growth on ACC as the only carbon and nitrogen source in a knockout study (5).

When growing on ACC as a sole C and N substrate, the supply of nitrogen is higher than necessary in relation to the carbon supply, and thus, most of the resulting ammonia (up to 57.8%) is exported again. The remaining portion is utilized in the production of L-glutamate, L-glutamine, carbamoyl phosphate, and L-asparagine. L-glutamate participates in the synthesis of all other amino acids either directly or through intermediates, including L-glutamine and L-asparagine. The amino acids are used for protein synthesis, which is part of the biomass reaction encoded in the model. Figures S1 and S2 show the amino acid pathway maps with the chain of reactions that are involved in amino acid synthesis as implemented in the network reconstruction, following carbon and nitrogen flux.

In this section, we focus on the reconstruction of a pathway for the further utilization of the carbon compound 2-oxobutyrate from ACC hydrolysis.

In the automated draft model reconstruction, 2-oxobutyrate was already included as part of the isoleucine biosynthesis pathway (MetaCyc Id: ILEUSYN-PWY). Thus, we were looking for possible connections from intermediates of that pathway to the core carbon pathways (TCA cycle and gluconeogenesis).

One option to connect is from the L-isoleucine precursor 3-methyl-2-oxopentanoate (3MOP), which is also part of an L-isoleucine degradation pathway (MetaCyc Id: ILEUDEG-PWY), to 2-methylbutyryl-CoA, which is subsequently transformed into propionyl-CoA and acetyl-CoA. From there, following the 2-methylcitrate cycle I pathway (MetaCyc Id: PWY0-42), pyruvate and succinate can be produced, which establishes the required link to the core carbon metabolism. To get this pathway, only the first reaction in the L-isoleucine degradation pathway from 3MOP to 2-methylbutyryl-CoA (MetaCyc Id: 2KETO-3METHYLVALERATE-RXN) had to be added manually to the automated reconstruction, while the others were already present with an appropriate gene annotation.

Another option to connect 2-oxobutyrate to the core carbon metabolism is a more direct transformation of 2-oxobutyrate into propionyl-CoA (MetaCyc ID: RXN-7790), which is part of the 2-oxobutanoate degradation pathway (MetaCyc ID: PWY-5130). This reaction is annotated in MetaCyc with *Homo sapiens* genes.

Interestingly, both reactions are known to be catalyzed by a branched-chain α-keto acid dehydrogenase (BCKD) system (EC 1.2.1.25, MetaCyc ID: CPLX-7050). BCKD is a multienzyme complex encoded by four genes and is found in mammalian mitochondria as well as bacteria and plants (28). For the first option, the reaction from 3MOP to 2-methylbutyryl-CoA, MetaCyc includes an annotation with genes from *Bacillus subtilis*, while the second option, the reaction from 2-oxobutyrate to propionyl-CoA, is annotated with *Homo sapiens* genes. In *B. subtilis*, the genes of the BCKD complex are BfmBAA (2-keto-isovalerate dehydrogenase α subunit), BfmBAB (2-keto-isovalerate dehydrogenase β subunit), BfmBB (lipoamide acyltransferase), and PdhD (dihydrolipoyl dehydrogenase) (29). In *H. sapiens*, the genes are branched-chain α-keto acid dehydrogenase E1 component α subunit (BCKDHA), branched-chain α-keto acid dehydrogenase E1 component β subunit (BCKDHB), branched-chain α-keto acid dehydrogenase E2 component (DBT), and dihydrolipoyl dehydrogenase (DLD) (30).

**Fig 2:**
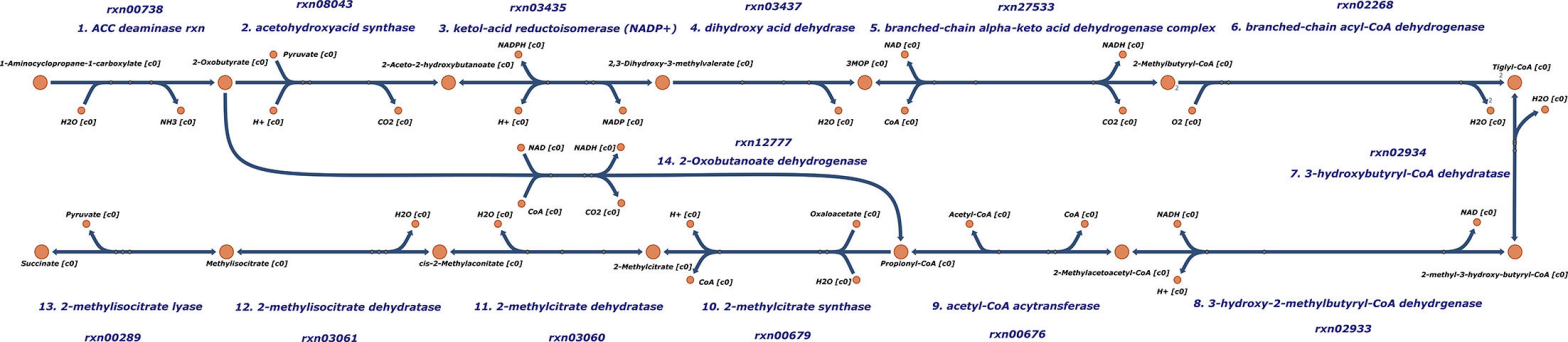
Pathway diagram for the reconstructed ACC consumption pathway, labeled with compound and reaction names according to the *P. dioscoreae* metabolic network model.

In order to find genetic evidence for the relevant reactions in *P. dioscoreae*, we ran a BLAST analysis of the relevant gene sequences against the *P. dioscoreae* genome (31). The results are shown in Table 2, with *P. dioscoreae* annotations from the RefSeq genome. The results provided sufficient evidence to add both reactions to the metabolic network reconstruction, with the gene annotations corresponding to the matches in Table 2. The reconstructed pathway is shown in Fig. 2.

**TABLE 2:** Blast results for branched-chain α-keto acid dehydrogenase genes against the *P. dioscoreae* (*Pd*) genome

Gene	Species	Best *Pd* match	Sequence identity	*e*-Value	*Pd* annotation
BfmBAA	*B. subtilis*	PDMSB3_RS34690	34%	9 · 10^−48^	Thiamine pyrophosphate-dependent dehydrogenase E1 component subunit alpha
BfmBAB	*B. subtilis*	PDMSB3_RS34685	44%	3 · 10^−86^	Alpha-ketoacid dehydrogenase subunit beta
BfmBB	*B. subtilis*	PDMSB3_RS07770	36%	6 · 10^−68^	2-Oxoglutarate dehydrogenase complex dihydrolipoyllysine-residue succinyltransferase
pdhD	*B. subtilis*	PDMSB3_RS07775	34%	2 · 10^−82^	Dihydrolipoyl dehydrogenase
BCKDHA	*H. sapiens*	PDMSB3_RS34690	34%	2 · 10^−37^	Thiamine pyrophosphate-dependent dehydrogenase E1 component subunit alpha
BCKDHB	*H. sapiens*	PDMSB3_RS34685	39%	3 · 10^−65^	Alpha-ketoacid dehydrogenase subunit beta
DBT	*H. sapiens*	PDMSB3_RS13205	28%	9 · 10^−39^	Dihydrolipoyllysine-residue acetyltransferase
DLD	*H. sapiens*	PDMSB3_RS33195	39%	7 · 10^−11^	NAD(P)/FAD-dependent oxidoreductase

Although both pathway alternatives have succinate as final metabolite, their energy efficiencies may be different due to differences in cofactor usage. It is already apparent in Fig. 2 that the longer pathway uses more cofactors than the short pathway, and indeed, when both options are available, the model optimization chooses the short pathway. This was also further clarified by testing the energy efficiency (in this and the following tests, the shortcut was blocked by a suitable constraint when running the model with the longer pathway was desired). We maximized the flux on ATP hydrolysis for growth on glucose and growth on ACC, using one or the other pathway alternative. On glucose, the model achieves 26.00 mmol ATP per mmol glucose consumed, whereas on ACC, it achieves 16.25 mmol ATP per mmol ACC along the shortcut pathway, and 14.38 mmol ATP per mmol ACC with only the longer pathway. However, if we scale these yields to the carbon content of the substrates (six carbon atoms for glucose, four for ACC), we get 4.33 mmol ATP per mmol carbon with glucose, 4.06 mmol ATP per mmol carbon on ACC with the short pathway, and 3.60 mmol ATP per mmol carbon with the long ACC pathway, meaning that at least the short ACC pathway is almost as ATP-efficient as the utilization of glucose. Although most of the carbon flux from ACC is routed along the short pathway in the optimal solution, there is still some small flux on a section of the long pathway for growth, from reactions 2 to 4, that constitute part of the L-isoleucine biosynthesis.

### The reconstructed ACC pathway is present in a range of leaf-associated bacteria

Having reconstructed a pathway for ACC consumption in *P. dioscoreae*, we next addressed the question of how widespread this pathway is in other bacteria that have the ACC deaminase gene or are associated with leaves. For that, we did a sequence homology search of the genes in the reconstructed pathway (Fig. 2) against the genomes of a recently published library of 223 bacterial strains of the *Arabidopsis thaliana* leaf microbiome (22) with BLAST. This library does not include *P. dioscoreae*.

**Fig 3:**
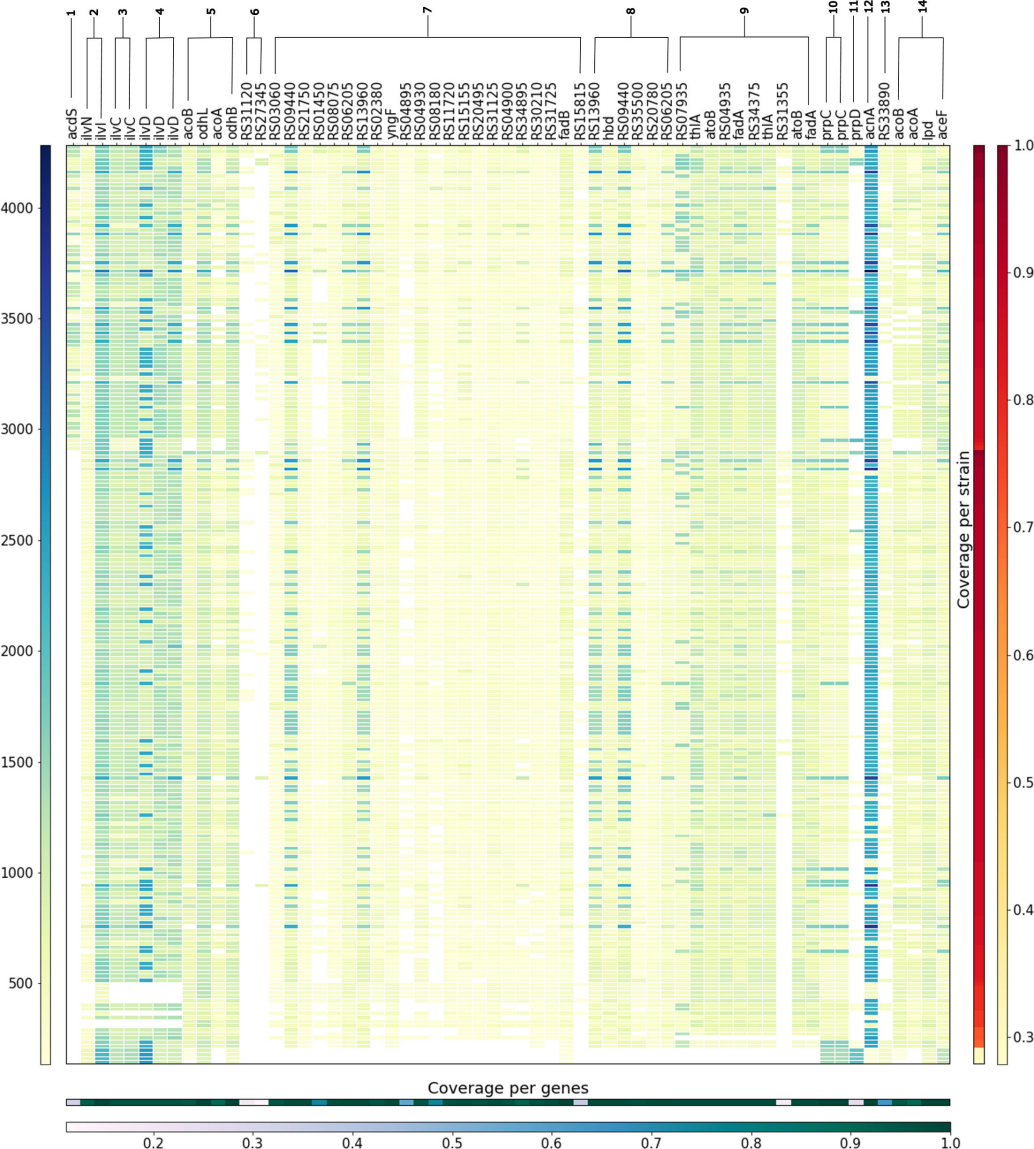
Blast analysis of genes in ACC consumption pathway. The columns correspond to the genes present in the pathway and the rows to the leaf bacterial strains. Numbers above the column titles refer to reaction numbers in the pathway diagram Fig. 2. The column to the right side of the heatmap shows the coverage per strain, that is, the percentage of genes in the pathway found in each strain. The row below the heatmap shows the coverage per gene, that is, the percentage of leaf strains that had that gene present. The genes or gene clusters are numbered in relation to the reactions they catalyze in Fig. 2. .

Figure 3 presents a heatmap with the scores for the blast analyses, where the best score was selected in case of multiple hits. The ACC deaminase (acdS) gene was found to be present in 33% of the strains. Two leaf strains were found to have all the genes from the reconstructed pathway present.

The genes for the reactions that were added in the pathway completion, related to the branched-chain α-keto acid dehydrogenase, were found in almost all of the strains: odhB, odhL, aceF, and lpd all have 100% coverage (PDMSB3_RS07770, PDMSB3_RS07775, PDMSB3_RS13205, and PDMSB3_RS33195), while acoA and acoB had 90% and 95% coverage, respectively (PDMSB3_RS34690 and PDMSB3_RS34685).

Other than the acdS gene, only a few of the pathway genes were found in less than 40% of the strains: the genes PDMSB3_RS27345 and PDMSB3_RS31120 encode branched-chain acyl-CoA dehydrogenase, which produces Tiglyl-CoA from 2-Methylnutyryl-CoA, and were found only in 11% or 17% of the strains, respectively. This reaction is only relevant for the long variant of the proposed ACC pathway, which may thus be less widespread than the short variant.

The gene prpD (PDMSB3_RS20965), which encodes for the 2-methylcitrate dehydratase, was found in 26% of the strains and appears to be the least commonly found of the genes that would be required for both ACC pathway alternatives.

Given an overall coverage of 87.9% of the ACC pathway genes within the considered strain library, we conclude that these genes are quite widespread in leaf-associated bacteria, even if the specific reactions proposed here may be used in a narrower subset.

It is also noticeable that the genes related to L-isoleucine biosynthesis (reactions 2–4) are well conserved, with an average coverage of 96% and a standard deviation of 1.8%. On the other side, although the L-isoleucine degradation (reactions 5–9) has an average coverage of 88%, it has a deviation of 23%, since the majority of genes are present in this pathway, including most of the genes with low coverage, where three genes have a coverage under 20%, four more from 30 to 80%, and the rest from 90 to 100%. Finally, the 2-methylcitrate cycle I pathway (reactions 10–13) has an average coverage of 78% and 32.9% of standard deviation, with three genes with 100% coverage, but two with 26% and 64%.

Data set S4 includes more details, like the *e*-values and the percent identity for all the hits found.

### ACC acts as a dual nitrogen and carbon source in combination with other sugars

While the preferred carbon sources of *P. dioscoreae* are simple C4 sugars, it also grows on glucose and ACC (3). Using the proposed metabolic network model, we investigated the possible interactions between different carbon sources and ACC as well as the difference between ACC and ammonium as a nitrogen source for growth and biomass yield.

Using flux balance analysis with the biomass reaction as the objective function, biomass yields were calculated for carbon uptake with glucose, succinate, malate, or ACC as a carbon source, with either ammonium or ACC as a nitrogen source (Table 3).

**TABLE 3:** Model-predicted biomass yields on different carbon sources (gDW per mmol carbon) with ammonium or ACC as a nitrogen source

Carbon source	Yield with NH4	Yield with ACC
Glucose	0.0170	0.0168
Succinate	0.0144	0.0157
Malate	0.0114	0.0140
ACC	0.0132^*a*^−0.0153^*b*^	0.0132^*a*^−0.0153^*b*^

^*a*^ Results using the long pathway.

^*b*^ Results using the shortcut pathway.

**Fig 4:**
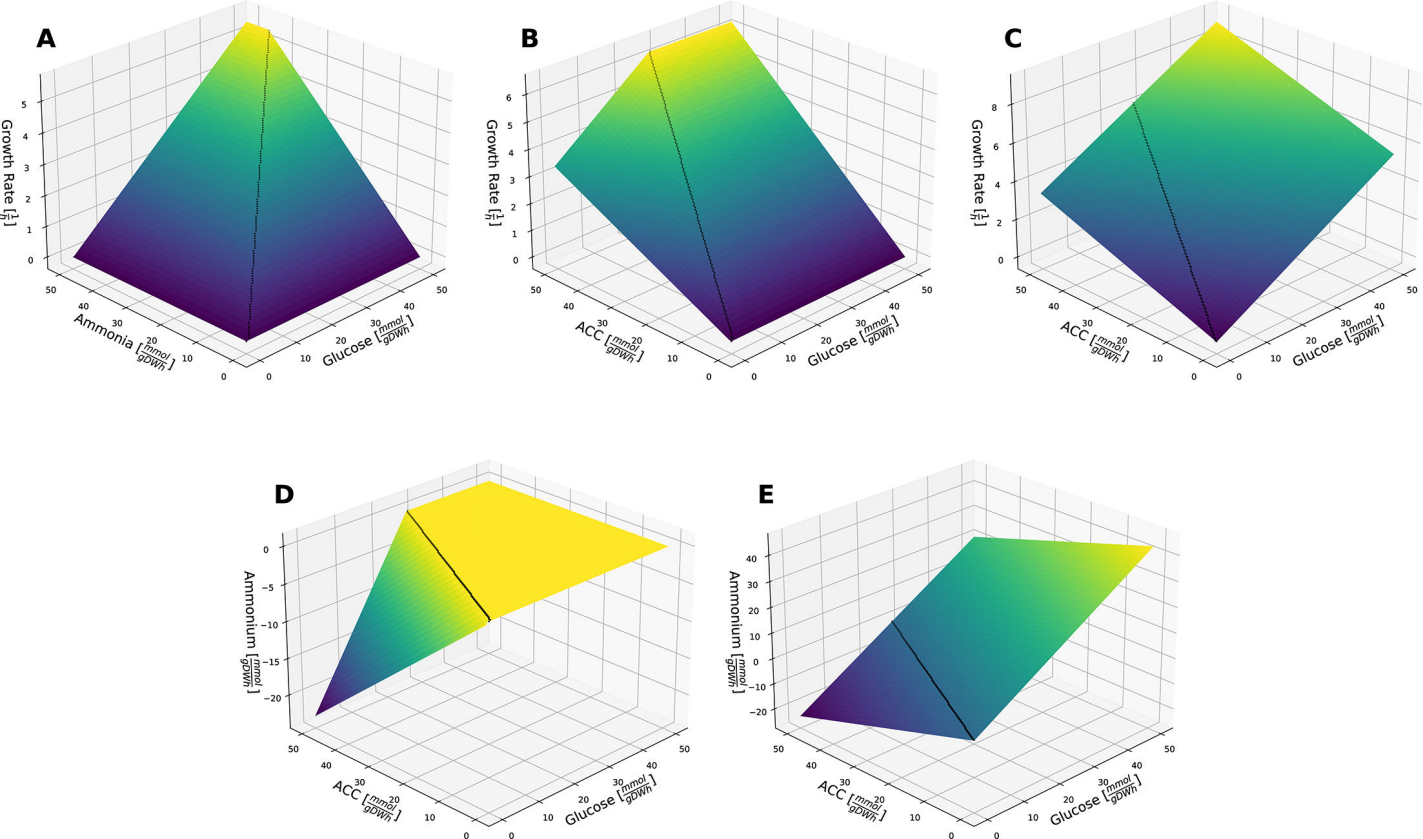
Phenotypic phase planes. (**A**) Growth rate with varying constraints for glucose and ammonium uptake. (**B**) Growth rate with varying constraints for glucose and ACC uptake, without ammonium. (**C**) Growth rate with varying constraints for glucose and ACC uptake, with unconstrained ammonium flux. (**D**) Ammonium uptake with the same conditions as for panel **B**. (**E**) Ammonium uptake with the same conditions as for panel **C**.

To further compare the impact of ACC as a nitrogen source with different carbon substrates, PhPPs were computed for three different conditions (Fig. 4).

With glucose and ammonium (Fig. 4A), we can see two zones of different metabolic constraints, represented by the differently inclined planes. With a high ammonium uptake constraint, growth is constrained by glucose and thus is carbon-limited, whereas with a high glucose uptake constraint, growth is constrained by ammonium and thus is nitrogen-limited. The line where the two planes meet indicates an optimal balance of about 0.85:1 ammonium to glucose uptake flux, at which both substrates are limiting.

With glucose and ACC, but no ammonium (Fig. 4B), there are two corresponding zones; however, there are important differences from the case with ammonium as a nitrogen source. First, in the zone where glucose is the limiting substrate, increasing the ACC uptake constraint still increases the growth rate. Since growth is actually carbon-limited, it can still be increased by the carbon content of ACC. Second, the threshold of switching between nitrogen- and carbon-limited growth is shifted toward an increased ACC flux, with a ratio of around 1:0.58 ACC to glucose. Correspondingly, in the zone of carbon-limited growth, cleavage of ACC leads to surplus ammonium, which would be secreted by the metabolic network (Fig. 4D).

Corresponding to the optimal C/N ratio from the analysis with glucose and ammonium, for every 6 units of carbon, 0.85 units of nitrogen are needed. ACC as a carbon source provides 4 units of carbon, for which 0.56 units of nitrogen would be needed to get the optimal ratio. However, ammonium with one nitrogen atom is a direct product of the ACC deaminase reaction, yielding an excess of 0.44 units of nitrogen which will be secreted by the model if fed with ACC alone. When, in addition to ACC, a sufficient amount of glucose is present to metabolize the nitrogen provided by ACC deamination, the model becomes nitrogen-limited, and there is no secretion of ammonium anymore.

Figure 4C shows the changes compared to the previous case when ammonium is unconstrained. In that case, growth is always carbon limited; thus, there are no different planes apparent in the diagram. Nevertheless, we can see the effect of ACC by examining the net ammonium flux, which shifts from negative (secretion of ammonium cleaved from ACC) to positive (uptake of extra ammonium) at exactly the same threshold where growth switches from carbon limited to nitrogen limited in the case without ammonium. This threshold is represented by a black line in Fig. 4C and E.

### Growth curves on glucose and ACC validate model-predicted biomass yields

*P. dioscoreae* MSB3 was grown in different configurations of glucose, ACC, and ammonium, that were compared against model predictions by normalizing to the glucose and ammonium case. As expected, different cell densities were reached in media supplied with different carbon and nitrogen sources. The OD values measured in lag-phase from highest to lowest were as follows: glucose + ammonium + ACC > glucose + ammonium > glucose + ACC > ACC + ammonium = ACC. Another difference we detected was the growth rate on different substrates. Msb3 grown with glucose + ammonium and glucose + ammonium + ACC showed the fastest increase in OD, corresponding to the highest growth rate, while ACC or ACC + ammonium showed the slowest growth rate. Msb3 on glucose + ACC grew at an intermediate growth rate (Fig. S3).

As further exploration on *P. dioscoreae* metabolic capacities, it was grown on malate and on succinate media, with ammonium as a nitrogen source. In both cases, similar OD values were achieved, ranked between the cases with glucose + ACC and ACC alone. Interestingly, in both cases, it grew faster than all other configurations (Fig. S3).

The optical densities reached in stationary phase give us an indication of biomass yield on the different substrates, which can be directly compared to the biomass reaction flux in an FBA solution of the metabolic network model. The relative yields, normalized to the optical densities with glucose + ammonium as a substrate, are compared against accordingly normalized model predictions in Table 4, where model 1 refers to the short ACC pathway with the reaction from 2-oxobutyrate to propionyl-CoA, while model 2 refers to the long pathway without that reaction. (These results can be seen graphically in Fig. S4, and the absolute values of experimental and FBA results are presented in Table S2 and Data set S5.)

**TABLE 4:** Comparison of optical density from growth experiments against model biomass flux predictions^*a*^

Substrates (2 mM each)	Normalized OD (experiment)	Normalized biomass (model 1)	Normalized biomass (model 2)
Glucose, NH_3_	1.0195 ± 0.1938	1.0	1.0
ACC	0.5075 ± 0.0832	0.5533 (*P* = 0.58)	0.4681 (*P* = 0.64)
ACC, NH_3_	0.4949 ± 0.0720	0.5533 (*P* = 0.42)	0.4681 (*P* = 0.71)
Glucose, ACC	0.9382 ± 0.1755	1.3637 (*P* = 0.02)	1.3637 (*P* = 0.02)
Glucose, ACC, NH_3_	1.3882 ± 0.2270	1.7471 (*P* = 0.12)	1.6641 (*P* = 0.23)
Malate, NH_3_	0.7145 ± 0.0934	0.3807 (*P* = 0.00)	0.3807 (*P* = 0.00)
Succinate, NH_3_	0.7472 ± 0.1097	0.5126 (*P* = 0.03)	0.5126 (*P* = 0.03)

^*a*^ Model 1, short ACC pathway variant; model 2, long ACC pathway variant. *P*-values from a one-sample *z*-test.

In both experimental and model results, the yield on ACC (alone and with ammonium) is about half of the yield on glucose, and in those three scenarios, the model result is inside the range of the standard deviation for the experimental results; the statistical analysis did not show a significant difference between experimental measurements and model predictions. In the other glucose cases, the experimental yield with ACC and ammonium is about 30% higher than without ammonium, whereas the model predicted a 20% higher yield between these cases. While the difference between the experiments and model predictions is statistically significant for growth on glucose and ACC, the difference is not significant in the case with glucose, ACC, and ammonium (although with a smaller *P*-value than in the cases with a single carbon substrate). With malate and succinate, the model predicted that they would give lower or similar yield, respectively, as ACC; while for both cases, the experimentally observed yield was a bit higher than with ACC, although clearly not as high as with glucose.

## DISCUSSION

The presence of ACC deaminase is a key trait in plant growth promotion, as it has a direct impact in decreasing ACC levels and thus ethylene levels in plants (9). While ACC deaminase (*acdS*) has been more studied in plant seed and root systems, bacteria with the gene have been found in the phyllosphere of different plants, including rice (32) and Antarctic vascular plants (33). Such bacteria have also been applied in the seeds of tomato plants, from where they later colonized the leaves (5, 34). In a past study, the *acdS* gene was found with over 84% sequence identity in 27 rhizobacterial strains out of 233 analyzed from 30 different sites (35). Here, we found genes similar to *acdS* with 25.5%–88.5% identity, and *e*-values from 6.5 · 10^−11^ to 0, for 74 strains out of 223 in a recently published genome collection of the phyllosphere (22).

ACC deaminase cleaves ACC into ammonium and α-ketobutyrate, thus possibly acting as both nitrogen and carbon source for the bacteria. While it has been shown experimentally that *P. dioscoreae* can indeed grow on ACC as the only nitrogen and carbon source (5), the pathway for the utilization of α-ketobutyrate was not yet known. Here, we present a new metabolic pathway integrated into a genome-scale metabolic network reconstruction, which links ACC to succinate and thus explains the carbon utilization from ACC. A key step in the reconstructed pathway is the BCKD system, which has two possible reaction mechanisms, leading to a short and a long alternative for the ACC pathway. Sequence similarity analysis of the *P. dioscoreae* genome showed significant hits against all subunits of this enzyme complex, supporting the addition of the associated reactions to the pathway.

Beyond ACC deaminase, in the overall pathway, the reactions related to the L-isoleucine biosynthesis are well conserved, with a high presence of all the corresponding genes through the species library. This is not surprising, as L-isoleucine is an amino acid and necessary for growth. On the other hand, other sections of the long pathway are not as well conserved, with some genes having low presence overall, while the shortcut reaction has full coverage. This suggests a preference for the shortcut pathway, which is the more efficient one, whereas the long pathway, less efficient, has fewer conserved genes. Nonetheless, both pathways share reactions 10–13, from the 2-methylcitrate cycle I pathway, where the gene associated to reaction 11 has a 26% coverage. An alternative to this pathway is the 2-methylcitrate cycle II pathway (MetaCyc ID: PWY-5747), where reaction 11 is separated in two reactions catalyzed by different genes. Other ACC-consuming organisms could have these alternative genes instead of the one found in *P. dioscoreae* and present in the current model.

Comparing our metabolic model with microbial growth curves, both show similar ratios of growth on ACC versus growth on glucose and ammonium, thus validating the relative efficiencies of carbon assimilation from glucose vs. carbon assimilation from ACC predicted by our model.

As was to be expected from a metabolic model analyzed with FBA, the biomass yield is predicted to become higher if both carbon sources are added. However, this is not confirmed by the experimental data, where instead a similar yield as with only glucose was observed for growth on ACC and glucose. Frequently, such mismatches can be explained by mechanisms that are not accounted for by FBA, such as enzyme activation and capacity limits, regulation of gene expression, or substrate prioritization (36, 37). In particular, metabolic model analysis with FBA is not always able to take prioritization of different alternative substrates into account (catabolite repression), which is commonly observed for microorganisms. While in some cases, FBA may correctly predict switches between alternative carbon sources, in other cases, such as glucose-lactose diauxie, additional regulatory constraints or other model extensions may need to be added for a correct prediction of catabolite repression (38).

Looking for a possible regulatory mechanism in this case, it has been noted that in some bacteria with ACC deaminase, the corresponding gene is regulated by a leucine-responsive regulatory protein (LRP), located upstream of the start of the *acdS* gene, and transcribed in the opposite direction (9, 39). Of the 27 rhizobacteria with *acdS* mentioned above, the LRP gene has been found in 17 (35), indicating a somewhat widespread coincidence of these two genes. In the *P. dioscoreae* genome, an Lrp/AsnC ligand-binding domain-containing protein can be found 164 base pairs upstream of the *acdS* gene, indicating that this regulation is also active in this strain.

Since 2-oxobutyrate is a precursor of branched-chain amino acids, such as leucine, the regulation represents a negative feedback on the *acdS* gene: when leucine accumulates, it binds to the LRP octamer, causing it to dissociate into inactive dimers, shutting down further transcription of *acdS*, thus ensuring the synthesis of ACC deaminase only when it is needed (9, 39).

Thus, differences between experimental data and model predictions could be due to a downregulation of the ACC pathway when other carbon sources are available. This hypothesis is also consistent with carbon assimilation from glucose being more efficient than from ACC, which would favor a form of catabolite repression if both substrates are available. Also, such an effect indicates that uptake of ACC is relevant as a possible nutrient source for the bacteria instead of only being a means to control the plant’s ethylene metabolism.

## Data Availability

All data generated or analyzed during this study are included in this published article and its supplemental files. The SBML model has been submitted to the BioModels database (https://www.biomodels.org) with model ID MODEL2604290001. Jupyter notebooks for model analysis have been made available at https://github.com/mosys-univie/p_dioscoreae_gsmm.

## SUPPLEMENTAL MATERIAL

Data set S1 msystems.00514-26-s0001.txt Metabolic network reconstruction in SBML format, with ACC medium applied.

Data set S2 msystems.00514-26-s0002.xlsx List of curated reactions.

Data set S3 msystems.00514-26-s0003.html MEMOTE report.

Data set S4 msystems.00514-26-s0004.xlsx BLAST results for ACC pathway in the phyllosphere.

Data set S5 msystems.00514-26-s0005.xlsx Growth culture experimental results.

Supplemental material msystems.00514-26-s0006.pdf Supplemental tables and figures.

ASM does not own the copyrights to Supplemental Material that may be linked to, or accessed through, an article. The authors have granted ASM a non-exclusive, world-wide license to publish the Supplemental Material files. Please contact the corresponding author directly for reuse.
