# Supplementary material for: A metabolic model of the leaf-associated bacterium Paraburkholderia dioscoreae links the ethylene precursor ACC to core carbon pathways
Source: mSystems. 2026 Jul 13;11(8):e00514-26. doi: 10.1128/msystems.00514-26 (PMC13491717; doi:10.1128/msystems.00514-26)
Supplement: Supplementary file 1 [file msystems.00514-26-s0006.pdf]

Supplementary material: "A metabolic model of the leaf-associated bacterium *Paraburkholderia dioscoreae* links the ethylene precursor ACC to core carbon pathways"

Pablo Rolle Hannah Auricht Andreea Marcut
Cristina Lopez-Hidalgo Wolfram Weckwerth Steffen Waldherr[1]

[1] University of Vienna, Dept. of Functional and Evolutionary Ecology, Vienna, Austria, steffen.waldherr@univie.ac.at

Table 1: Flux bounds on exchange reactions according to medium composition.

Metabolites	Flux Bound	Metabolites	Flux Bound	Metabolites	Flux Bound
H2O	-1000	Zn2+	1000	Co2+	-1000
O2	-10	Sulfate	1000	K+	-1000
Phosphate	-1000	Cu2+	1000	Ni2+	-1000
NH3	-10	Ca2+	1000	Mg	-1000
D-Glucose	-5	H+	1000	Na+	-1000
Mn2+	-1000	Cl-	1000	Fe3+	-1000

Table 2: Experimental vs model

Substrates (2 mM each)	OD (exp.)	biomass (model 1) $[\frac{1}{h}]$	biomass (model 2) $[\frac{1}{h}]$
Glucose, NH3	2.5104 ± 0.3454	0.1835	0.1835
ACC	1.2510 ± 0.1260	0.1015	0.0859
ACC, NH3	1.2213 ± 0.0842	0.1015	0.0859
Glucose, ACC	2.3097 ± 0.3077	0.2502	0.2502
Glucose, ACC, NH3	3.4196 ± 0.3471	0.3206	0.3054
Malate, NH3	1.7639 ± 0.0385	0.0699	0.0699
Succinate, NH3	1.8417 ± 0.1282	0.0941	0.0941

Table 1 shows an example of glucose minimal media used in the model. Flux bounds correspond to the lower boundary for the metabolites exchange reactions, which can be modified to represent different configurations.

Table 2 shows the average ± standard deviation of the absolute values, in stationary face, from the experimental results in OD600, and the absolute values of growth rate $[\frac{1}{h}]$ obtained by the model, using the short ACC pathway (model 1) and the long pathway (model 2).

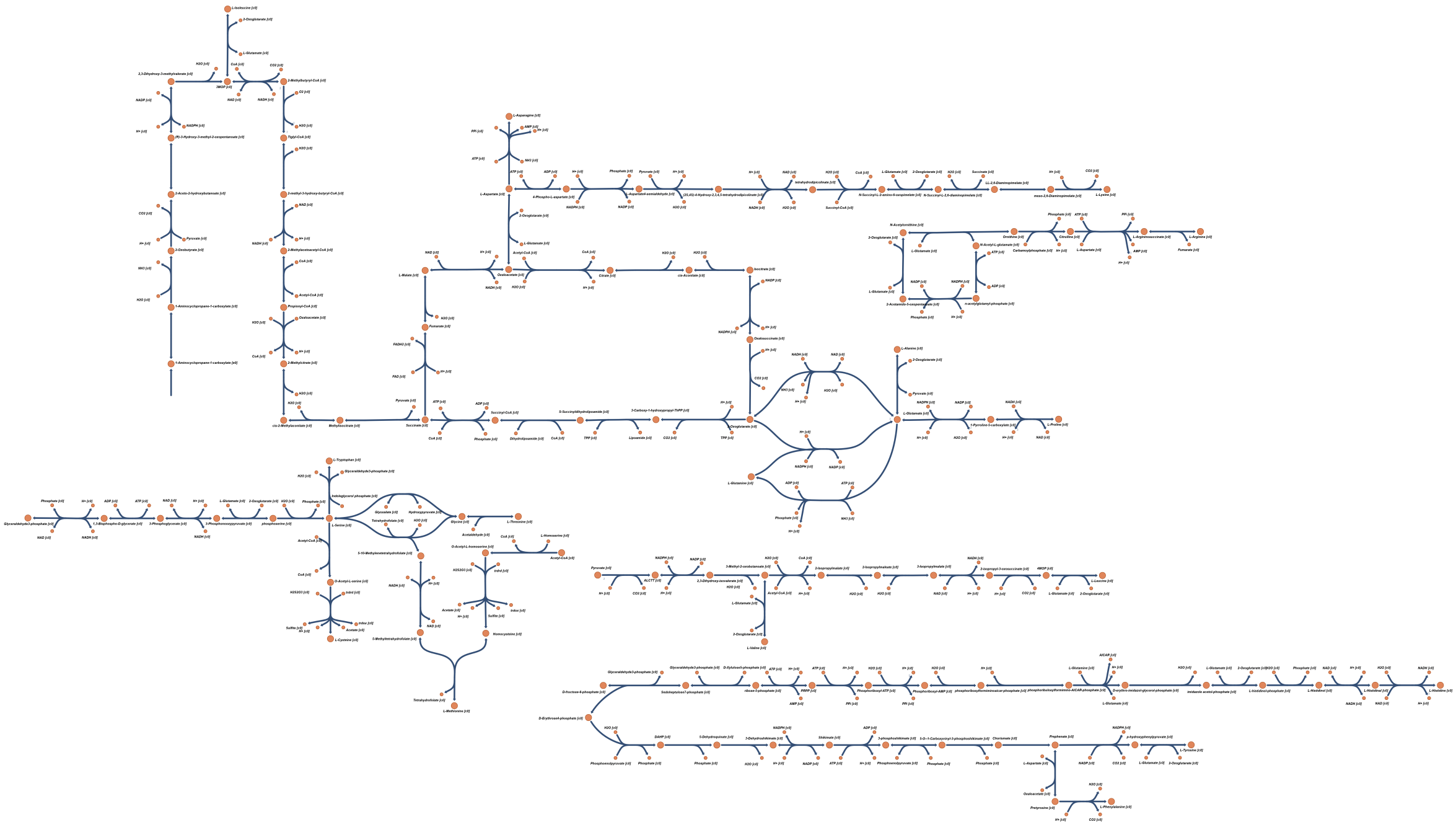

Figure 1: Aminoacids production from ACC and central carbon compounds following carbon flux, labelled with compound names according to the *P. dioscoreae* metabolic network model.

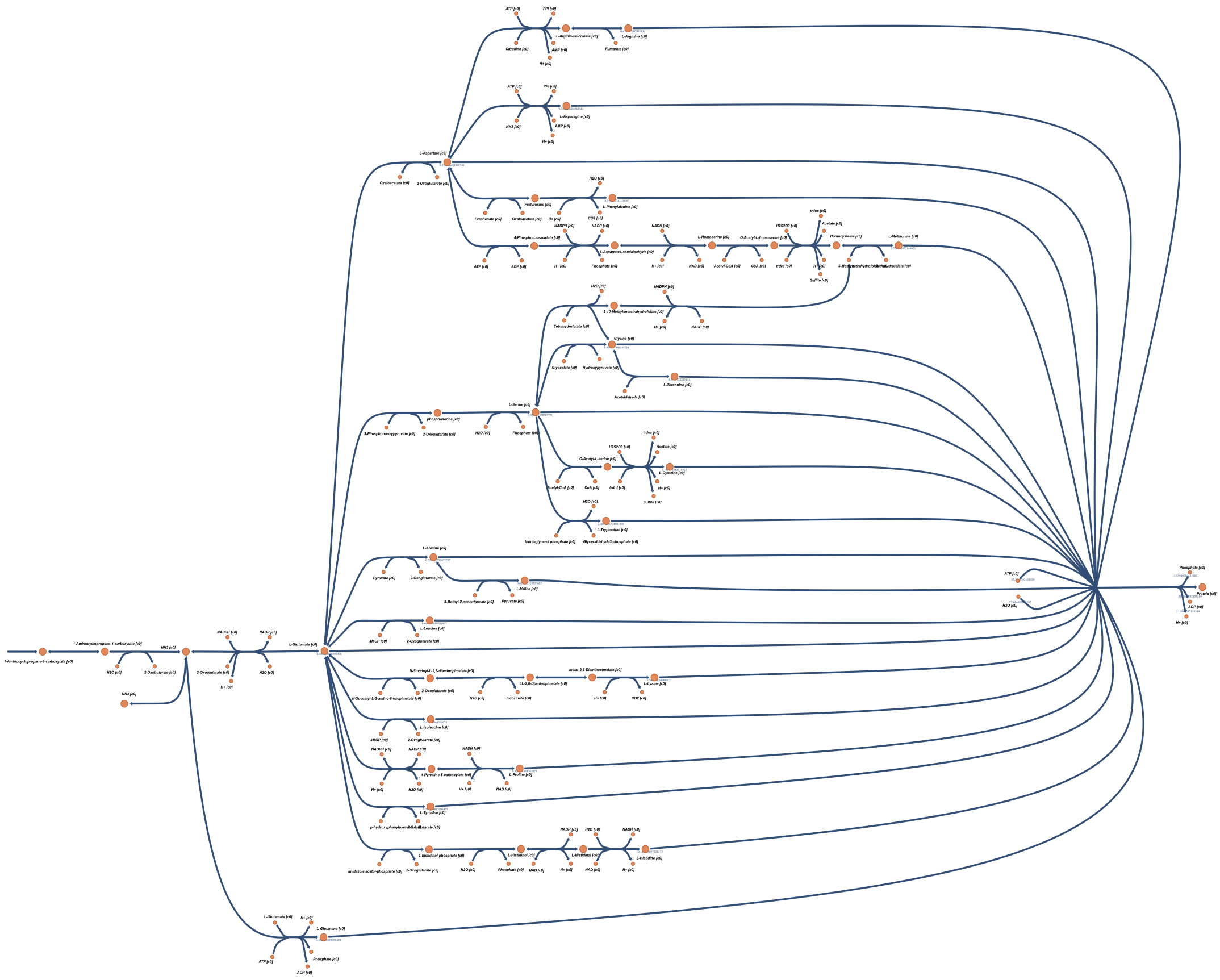

Figure 2: Aminoacids production from ACC following nitrogen flux, labelled with compound names according to the *P. dioscoreae* metabolic network model.

Figures 1 and 2 show the production of aminoacids. Figure 1 connects from ACC and central carbon compounds to the different aminoacids following the carbon flux through the metabolism, showing too the TCA cycle. Figure 2 connects from the ammonium obtained from ACC to the protein production reaction, following the main flow of nitrogen through the network. Figures were built using Escher Tools.

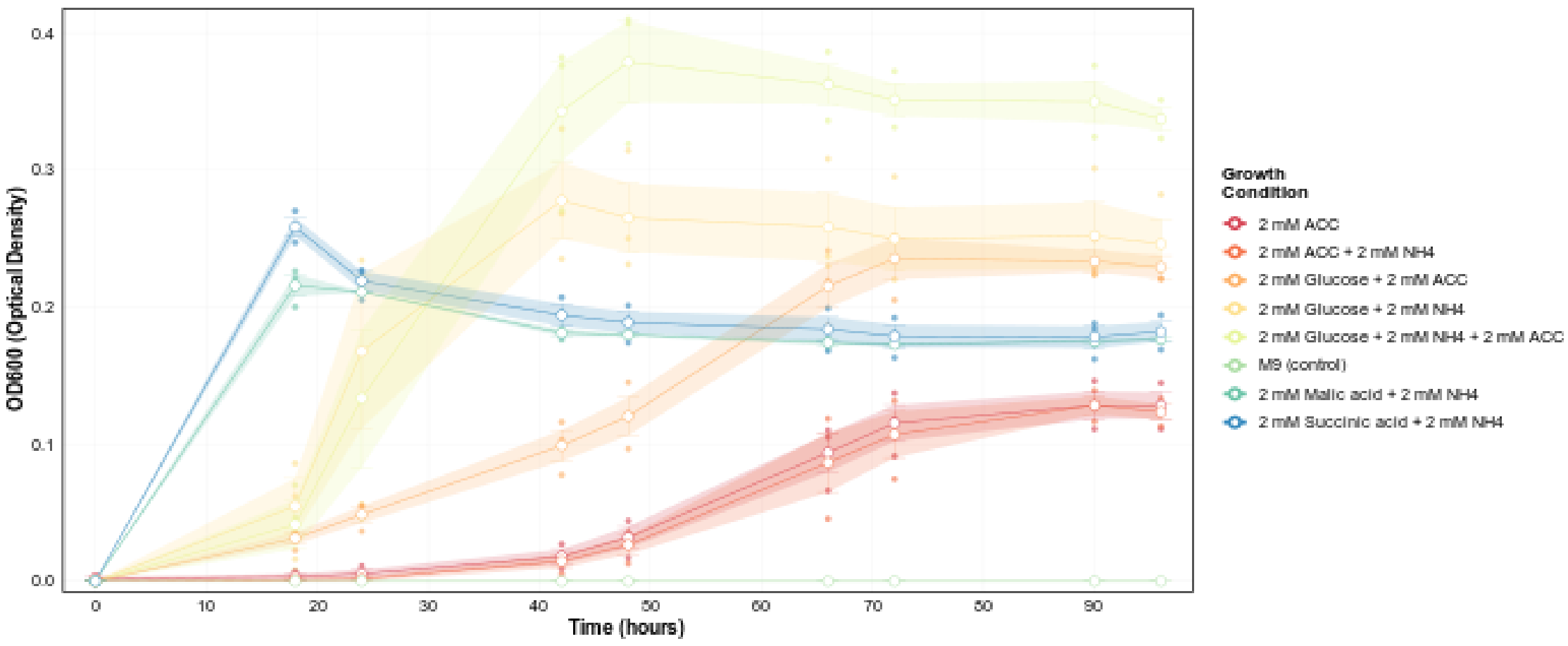

Figure 3: Bacterial growth curves under different nutritional conditions.

Figure 3 shows growth of Msb3 cultures monitored by OD_{600} measurements over 96 hours, under eight different carbon and nitrogen source combinations. Lines represent mean values from biological triplicates (n=3) with standard error bars. Shaded areas indicate 95% confidence intervals. Individual data points from each replicate are shown as semi-transparent circles.

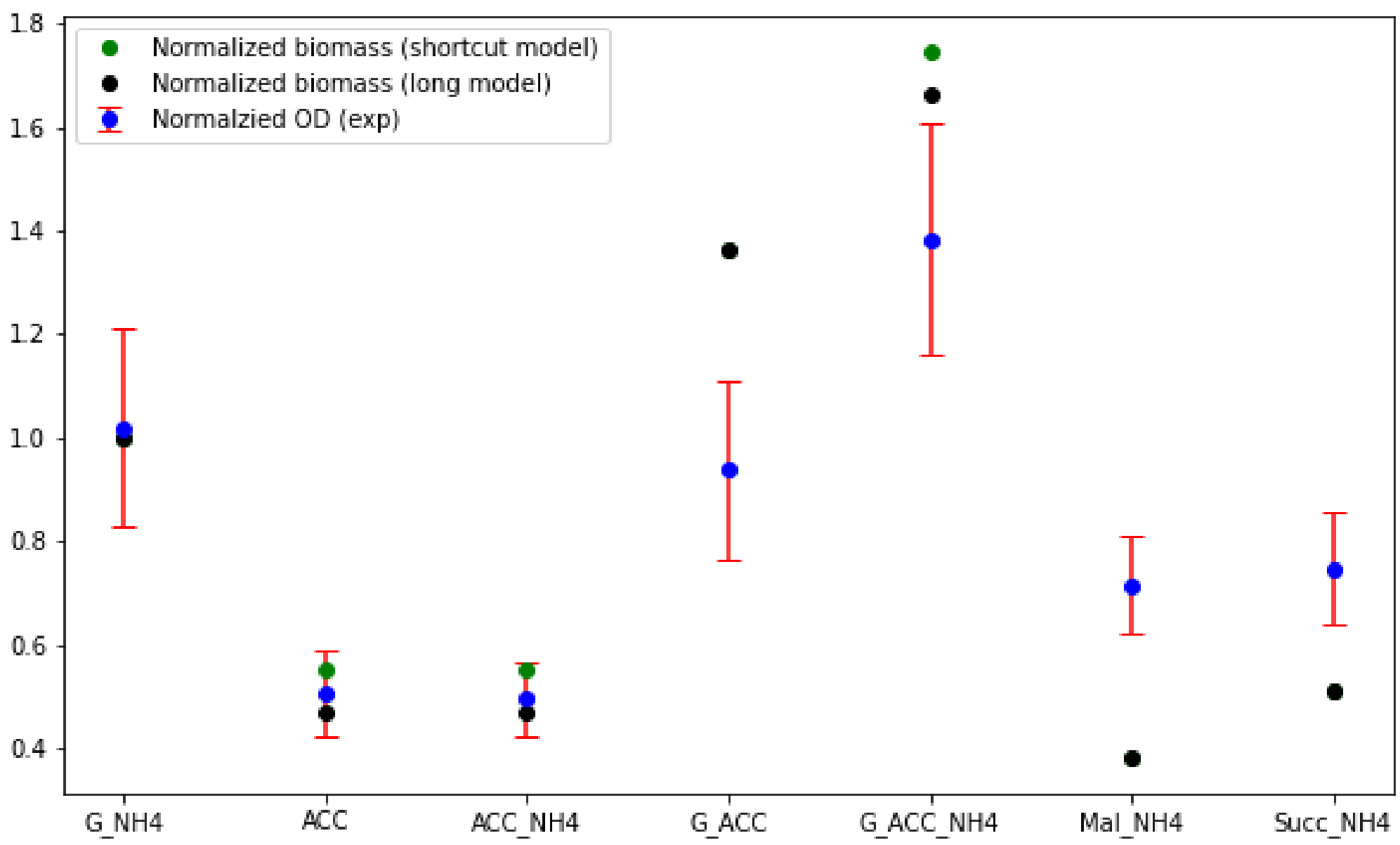

Figure 4: Model comparison to experimental results, normalized to the glucose and ammonium case.

Figure 4 shows experimental and model results normalized against the case of growth with glucose and ammonium. For the experimental data, the OD_{600} values in steady state of one case were randomly paired to the ones of glucose and ammonium with 1000 iterations, from where the mean and standard deviation were obtained, presented as blue dot and red lines. The green and black dots are the normalization values for the model results, using the shortcut and the long ACC pathway, respectively.
